# Comparison of Cortical Autonomic Network-Linked Sympathetic Excitation by Mueller Maneuvers and Breath-Holds in Subjects With and Without Obstructive Sleep Apnea

**DOI:** 10.3389/fphys.2021.678630

**Published:** 2021-05-26

**Authors:** Keri S. Taylor, Daniel A. Keir, Nobuhiko Haruki, Derek S. Kimmerly, Philip J. Millar, Hisayoshi Murai, John S. Floras

**Affiliations:** ^1^University Health Network and Mount Sinai Hospital Department of Medicine, Toronto General Hospital Research Institute and the Department of Medicine, University of Toronto, Toronto, ON, Canada; ^2^School of Kinesiology, The University of Western Ontario, London, ON, Canada; ^3^Division of Kinesiology, Faculty of Health, Dalhousie University, Halifax, NS, Canada; ^4^Department of Health and Nutritional Sciences, University of Guelph, Guelph, ON, Canada

**Keywords:** breath hold, sleep apnea, sympathetic nerve activity, Mueller maneuver, functional magnetic resonance imaging (fMRI)

## Abstract

In healthy young volunteers, acquisition of blood oxygen level-dependent (BOLD) magnetic resonance (MR) and muscle sympathetic nerve (MSNA) signals during simulation of obstructive or central sleep apnea identified cortical cardiovascular autonomic regions in which the BOLD signal changed synchronously with acute noradrenergic excitation. In the present work, we tested the hypothesis that such Mueller maneuvers (MM) and breath-holds (BH) would elicit greater concomitant changes in mean efferent nerve firing and BOLD signal intensity in patients with moderate to severe obstructive sleep apnea (OSA) relative to age- and sex-matched individuals with no or only mild OSA (Apnea Hypopnea Index, AHI, <15 events/h). Forty-six participants, 24 with OSA [59 ± 8 years; AHI 31 ± 18 events/h (mean ± SD); seven women] and 22 without (58 ± 11 years; AHI 7 ± 4; nine women), performed a series of three MM and three BH, in randomly assigned order, twice: during continuous recording of MSNA from the right fibular nerve and, on a separate day, during T2^∗^-weighted echo planar functional MR imaging. MSNA at rest was greater in those with OSA (65 ± 19 vs. 48 ± 17 bursts per 100 heart beats; *p* < 0.01). MM and BH elicited similar heart rate, blood pressure, and MSNA responses in the two cohorts; group mean BOLD data were concordant, detecting no between-group differences in cortical autonomic region signal activities. The present findings do not support the concept that recurring episodes of cyclical apnea during sleep alter cortical or peripheral neural responsiveness to their simulation during wakefulness by volitional Mueller maneuvers or breath-holds.

## Introduction

Initiated by occlusion of the upper airway, obstructive sleep apnea (OSA) is characterized by recurring pauses in breathing, which, if sustained, beget progressive hypoxia and hypercapnia until arousal from sleep causing ventilation to resume. Each of apnea, hypoxia, hypercapnia, and arousal from sleep stimulates, reflexively and additively, synchronous clusters of increased sympathetic nerve firing. The latter interrupt, cyclically, the reductions in blood pressure and heart rate typical of normal sleep (Somers et al., 1995; Bradley and Floras, 2003; Floras, 2018). These acute disturbances induce, over time, increased peripheral chemoreceptor reflex sensitivity (Mateika et al., 2004; Mahamed et al., 2005), upward resetting of central sympathetic outflow during wakefulness, and thickening and thinning of specific gray matter constituents of a cortical autonomic network (CAN) engaged in the generation or modulation of cardiovascular autonomic tone (Taylor et al., 2016a, 2018; Floras, 2018). The latter can be considered one element of a broader human blood pressure regulating “connectome” converging on brainstem sympathetic motor units (Macefield and Henderson, 2019). The magnitude of efferent post-ganglionic muscle sympathetic nerve firing recorded during wakefulness is directly proportional to the frequencies of nocturnal breathing cessation and arousal from sleep; the magnitude of subsequent arterial oxygen desaturation; and structural changes within the CAN, specifically, left mid-cingulate cortex thickness and thalamic volume (Taylor et al., 2016a,b, 2018; Floras, 2018).

Application of independent component analysis to blood oxygen level-dependent (BOLD) contrast functional magnetic resonance imaging (fMRI) during wakefulness, during documented spontaneous normal breathing, identified spatially large and strong correlations between the strength of resting-state connectivity and the magnitude of muscle sympathetic nerve firing, expressed as burst incidence, i.e., the percentage of cardiac cycles accompanied by a pulse-synchronous discharge within several CAN nodes of the salience network, which is a cluster of spatially distinct brain regions including the left insular cortex, the right pregenual anterior cingulate cortex, the left temporo-parietal junction, the thalamus, and the cerebellum with temporally correlated spontaneous BOLD oscillations at <0.1 Hz that is engaged by autonomic challenges and homeostatic threats (Taylor et al., 2009). Connectivity within the paraventricular nucleus of the hypothalamus, periaqueductal gray, pons, and rostral ventral lateral medulla also correlated with sympathetic burst incidence. However, despite evident structural differences (Taylor et al., 2018), in this investigation, dual-regression analysis discerned no difference with respect to the strength of resting-state connectivity within these salience network regions between study participants with moderate or severe OSA, relative to matched subjects with no or only mild OSA (Taylor et al., 2016a). Importantly, these signals were acquired during wakefulness, in the absence of apnea.

In earlier experiments, in which BOLD fMR images and MSNA recordings were acquired in healthy young volunteers at rest and during two volitional interventions selected to simulate obstructive [Mueller maneuvers (MM)] or central sleep apnea [breath-holds (BH)] (Bradley et al., 2003; Kimmerly et al., 2013), we identified cortical and cerebellar cardiovascular autonomic regions in which the BOLD signal either increased (activation) or decreased (deactivation) profoundly and synchronously with acute sympathetic excitation. The greatest increases in such neural activity were evident in the right posterior and anterior insular cortices and in the dorsal anterior cingulate, fastigial, and dentate cerebellar nuclei, whereas signal intensity decreased in the left posterior insula and ventral anterior cingulate (Kimmerly et al., 2013).

The present purpose was to determine whether such acute neuroanatomical substrate-adrenergic outflow coupling differs quantitatively or qualitatively between cohorts of otherwise similar individuals documented to have, or not have, OSA. We tested the hypothesis that the simulation of obstructive or central sleep apnea by these two volitional interventions would elicit greater changes in efferent muscle sympathetic firing and BOLD signal activation or deactivation in those subjects with OSA.

## Materials and Methods

### Participants

We recruited, primarily by advertisement, middle-aged men and women, self-identified as being in good health, who were agreeable to participating in research requiring overnight polysomnography, a daytime physiological recording session, and a functional MRI session. These investigations were approved by the Research Ethics Board of the University Health Network, in accordance with principles articulated in the Declaration of Helsinki. Informed written consent was obtained from all subjects in advance of their participation.

Cognizant of the high prevalence of asymptomatic and therefore unrecognized OSA in the general population (Kasai et al., 2012; Franklin and Lindberg, 2015) and to mitigate the commonplace biases risked by selecting as OSA patients only those identified as such after symptoms prompted referral for sleep studies and recruiting as control subjects individuals who self-reported freedom from OSA, we performed polysomnography first, then categorized participants as having either moderate to severe OSA (Apnea–Hypopnea Index; AHI ≥ 15 events/h) or no or mild OSA (AHI < 15 events/h) (Berry et al., 2012).

Each individual completed a standard MRI prescreening questionnaire, to ensure they could be exposed safely to a high magnetic field. Screening also excluded from study smokers, pregnant women, or patients treated for OSA or known to have central sleep apnea, as well as individuals with atrial fibrillation or a medical history of heart failure, myocardial infarction, frequent atrial or ventricular ectopy, kidney disease, Raynaud’s disease, autonomic neuropathy, drug-resistant hypertension, neurological disorders, or chronic back pain. Prescribed medications were continued to maintain clinical stability.

Two experimental sessions (physiological and MRI) were performed at the same time of day, within 2 weeks of each other, 2–3 h after a standard light meal. Subjects were instructed to abstain from alcohol and caffeine for at least 12 h before these sessions. Resting-state connectivity data, acquired in the majority of these participants, has been previously reported (Taylor et al., 2016a).

### Polysomnography

On the evening before the sleep laboratory study, the Epworth Sleepiness Scale (ESS) questionnaire (Johns, 1994) was administered by a technician unaware of this protocol’s purpose. Subjects then underwent overnight polysomnography using standard techniques and scoring criteria for sleep stages and arousals from sleep (Bonnet et al., 1992). Thoraco-abdominal movements and tidal volume were measured by respiratory inductance plethysmography. Airflow was measured by nasal pressure cannulae and arterial oxyhemoglobin saturation (SaO_2_) by oximetry. Mean and minimum SaO_2_ (minSaO_2_) were recorded. The oxygen desaturation index (ODI) was calculated as the frequency with which SaO_2_ fell by ≥3% during each hour of sleep (American Academy of Sleep Medicine., 1999). Apneas and hypopneas were scored according to the American Academy of Sleep Medicine criteria (Berry et al., 2012). OSA severity was graded by a continuum with an AHI < 5 events/h of sleep classified as no sleep apnea; AHI 5–15, mild OSA; AHI 15–30, moderate OSA; and AHI > 30, severe OSA (American Academy of Sleep Medicine., 1999; Berry et al., 2012).

### Sympathetic and Hemodynamic Data Acquisition and Analysis

Physiological recordings were acquired in a quiet, temperature-controlled room. Heart rate (HR) was derived from Lead II of an electrocardiogram. Blood pressure was recorded continuously from the right hand index finger (Portapres, Finapres Medical Systems B.V., Netherlands) and at each minute from an upper arm cuff (Dinamap Pro 100, Critikon, Tampa, FL, United States; standard 23–33-cm adult cuff). Breathing was recorded using a pneumobelt connected to a pressure transducer. Multiunit recordings of post-ganglionic muscle sympathetic nerve activity were acquired using a unipolar tungsten microelectrode inserted percutaneously into a fascicle of the right common fibular nerve (Kimmerly et al., 2013; Taylor et al., 2016b). MSNA, blood pressure, and HR were acquired over two separate 5-min “Apnea Protocol” periods. Using the LabVIEW^®^ software platform (National Instruments, Austin, TX, United States), signals underwent analog-to-digital conversion for storage and analysis on a standard PC desktop. To evaluate task-stimulated efferent sympathetic outflow and to control for any between-subject variation in HR, MSNA was expressed conventionally as the cardiac frequency-independent measure of sympathetic discharge intensity, bursts per 100 heart beats, or burst incidence (BI).

### Apnea Protocol

Before data were collected, each subject participated in a standardized practice run to ensure familiarity with the protocol and the ability to follow verbal instructions (i.e., “the next BH will begin in 10 s…0.5, 4, 3, 2, 1, hold”) and then meet the target negative inspiratory pressure and maintain this over the required time period (Kimmerly et al., 2013).

**FIGURE 1:**
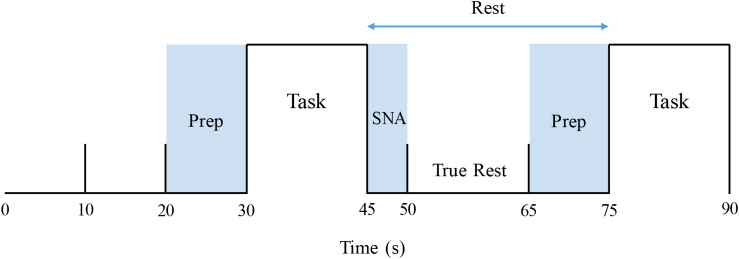
Stimulus paradigm, covering the first 1.5 min of an apnea protocol. Prep: verbal instruction period. Task: Mueller maneuver or breath-hold (order for each participant allocated at random). SNA: “Spill-over” period during which elicited sympathetic excitation returned to baseline. True rest: the 15-s period between these two intervals that was used to determine pre-intervention resting baseline values.

The identical apnea protocol was performed on 1 day within the physiology laboratory and on a second within the MR suite. On both occasions, participants breathed through a mouthpiece connected to a transducer that measured airway load pressure. To acquire baseline data, signals were recorded over at least 10 min of quiet supine rest with documented spontaneous breathing. The 5-min apnea sequence began with 30 s of rest, followed by 15 s of either the MM or 15 s of end-expiratory BH, assigned at random (Figure 1). For each participant, the task order during the two (MR and physiological) sessions was identical. Each sequence consisted of three 15-s MMs and three 15-s BHs. After recovery to baseline, the sequence was duplicated. For the MM, a stopper with a small air leak was inserted into the end of the mouthpiece. Subjects generated then sustained a negative pressure of −30 mmHg for 15 s. For the BH, task subjects were instructed to hold their breath for 15 s following a normal expiration. To ensure adherence with these endpoints and that the target inspiratory pressure was achieved and sustained throughout the MM, generated pressures were displayed continuously on a computer screen within subjects’ line of sight.

### Muscle Sympathetic Nerve Activity Acquisition Session: Statistical Analysis

Physiological data are expressed as means ± SD. Group differences were compared by unpaired Student’s *t*-test. BH and MM-stimulated changes were analyzed and compared, between cohorts, using a repeated-measure two-way ANOVA using SigmaStat 3.5 (Systat Software Inc., Chicago, IL, United States). For BHs, data acquired during the 15-s task were compared with baseline values. For MMs, due to the time delay preceding sympathetic excitation, and the carryover of such excitation after the end of the intervention, data during the 15 s of the task plus the first 5-s interval following this task were compared with baseline values. Tukey’s *post hoc* analysis was performed to estimate differences among means. A probability threshold of <0.05 was set for statistical significance.

### Magnetic Resonance Data Acquisition and Analysis

Magnetic resonance imaging (MRI) data were obtained while subjects lay supine within a 3-Tesla whole body scanner (HDxt 16.0 General Electric Health Care, Milwaukee, WI, United States) fitted with an eight-channel phased-array head coil. Foam pads were positioned on either side of the head to minimize its movement during the breathing tasks, and under the elbows and knees for participant comfort. An MRI-compatible vital sign monitor (Veris, Medrad Inc., Warrendale, PA, United States) recorded the ECG at rest and during anatomical data collection and end-tidal CO_2_ during the assigned breathing tasks. Headphones and MR-compatible goggles were worn to transmit instructions (i.e., “begin breath hold”) and to feedback visually adherence to the MM protocol, respectively. Gradient-echo echo-planar imaging-sensitive BOLD contrast, reflecting local field potentials (Ogawa et al., 1990), was used to identify cortical and cerebellar regions in which blood flow and/or metabolism changed from baseline during assigned tasks. Brain stem regions were excluded from study; it was assumed, from prior experience (Kimmerly et al., 2013), that movement artifact therein would confound precise localization of any induced BOLD signal changes.

A three-dimensional (3D) high-resolution anatomical scan of the whole brain (120 slices, 24_24-cm FOV, 256_256 matrix, 1.5_0.859_0.859 voxels) was acquired with a T1-weighted 3D spoiled gradient echo sequence (flip angle_45°, TE_5 ms, TR_25 ms). Functional MRI data were acquired with a T2^∗^-weighted echo planar imaging sequence (32 interleaved contiguous axial slices, FOV_20_20 cm, 64_64 matrix, 3.75_3.75_4.4-mm voxels, TE_40 ms, TR_2,000 ms). The scan was 5 min long, for a total of 150 frames. Each functional scanning period utilized the same apnea protocol described for the physiological testing session [i.e., two 5-min runs consisting of a 30-s rest followed by a 15-s apnea with a total of six blocks of apnea (three BH and three MM) and eight blocks of rest].

Magnetic resonance imaging data were analyzed with statistical parametric mapping software (SPM12, Wellcome Department of Cognitive Neurology) (Friston et al., 1995). The first three frames of each run (6 s) were discarded to allow for T1 equilibration effects. Next, subject data were corrected for slice timing collection and motion-related artifacts using SPMs Realign and Unwarp (which has been recommended when data are subject to task-correlated motion) (Jezzard and Clare, 1999; Andersson et al., 2001). Data were then co-registered to their anatomical scans and normalized to Montreal Neurological Institute (MNI) coordinates so that image volumes for all subjects were in the same three-dimensional space (Friston et al., 1995). A Gaussian smoothing kernel, set at 8 mm, was applied to allow better alignment of cortical anatomy across individuals. Data were high-pass filtered with a 90-s time constant to minimize low-frequency noise. Smoothed images then were de-trended for global signal intensity correction, to account for changes due to CO_2_ and/or perfusion variations.

Despite their practice sessions, task-correlated head motion was evident nonetheless in most subjects, regardless of cohort. To reduce confounding of image interpretation by such movement, any subjects with greater than 4 mm of translational (x, y, or z) and 4° of rotational (pitch, roll or yaw) motion were excluded.

Resulting data were analyzed utilizing a two-level statistical paradigm. The first-level analysis was used to identify within-subject differences in signal intensity between the particular apnea stimulus (MM or BH) and baseline. To accomplish this, a time course was constructed individually for each subject based on their MSNA response, as described in detail by Kimmerly (2013). For each apnea, the following measurements were acquired from the subjects’ physiological testing session: (1) MSNA delay: time from the beginning of each apnea (MM or BH) to the initiation of the MSNA burst and (2) MSNA duration. These values were measured for each BH and MM, across both 5-min runs of the apnea protocol (thus there were six MM and six BH measurements for delay and duration), in each individual subject. From these measurements, the average MSNA delay and duration were calculated for each subject for MM and BH separately. As the MSNA response persisted after the end of the apnea, the first 5 s of each subsequent period were not included when calculating pre-intervention rest data for the next task. Furthermore, as task instructions were given during the last 10 s of each rest period, that time also was excluded from resting value calculations. Thus, baseline, pre-intervention calculations were restricted to those “true rest” data acquired over the 15 s between the dissipation of prior sympathetic excitation and the verbal instructions for the next apnea (Figure 1).

Average delay and duration values combined with the true rest time periods were utilized to create individual fMRI regressors for each subject. These regressors were then convolved with the canonical hemodynamic response function and correlated with each subject’s fMRI time series for each voxel of the brain. Subject-specific contrast images containing whole-brain information related to both increase and decrease in BOLD signal for MM versus baseline and BH versus Baseline were constructed. These contrast images were utilized at the second level in a two-sample *t*-test to compare differences between groups (healthy control vs. OSA) for BH and MM separately. Contrasts were generated to examine BH > rest, rest > BH, MM > rest, and rest > MM. An uncorrected threshold of *p* < 0.001 and a minimum cluster size of 10 voxels were required to refute the null hypothesis.

## Results

### Participant Characteristics

Forty-six such individuals (average age 59), of whom 16 were women, completed all three tasks. Of these, 24 (seven women) were classified, following polysomnography, as having moderate to severe OSA [AHI, 31 ± 18 events per hour (mean ± SD)] and 22 (9 women) who had either no or mild sleep apnea (AHI, 7 ± 4 events per hour) were categorized as controls. Participants’ characteristics are presented in Table 1. As anticipated, mean values for both MSNA BI (65 ± 19 vs. 48 ± 17 bursts per 100 heart beats; *p* < 0.01) and the ODI (30 ± 21 vs. 11 ± 11 events per hour; *p* < 0.01) were greater in those with moderate to severe OSA, as compared with individuals with no or only mild OSA. Mean age, BMI, HR, and systolic blood pressure were similar, whereas diastolic pressure was significantly higher in those with OSA.

**TABLE 1:** Participant characteristics.

	Control (No or mild apnea)	OSA (Moderate or severe apnea)	
Characteristics	*n* = 22	*n* = 24	*p*-value
Age (years)	59 ± 8	58 ± 11	0.867
Men (*n*)	13	17	
AHI (events/h)	7 ± 4	31 ± 18	<0.001
BMI (kg/m^2^)	28 ± 5	31 ± 5	0.117
MSNA (bursts/100 HB)	48 ± 17	65 ± 19	<0.01
MSNA (bursts/min)	30 ± 11	42 ± 12	<0.001
HR (bpm)	59 ± 10	64 ± 9	0.240
SBP (mmHg)	122 ± 17	127 ± 13	0.179
DBP (mmHg)	64 ± 8	71 ± 10	<0.01
ODI	11 ± 11	30 ± 21	<0.01

*Mean ± SD.AHI, apnea–hypopnea index; BMI, body mass index; DBP, diastolic blood pressure; HB, heart beats; HR, heart rate; MSNA, muscle sympathetic nerve activity; ODI, oxygen desaturation index; OSA, obstructive sleep apnea; SBP, systolic blood pressure.*

After blinded analysis and extraction of those BOLD signal recordings that did not meet prespecified stringent head movement artifact exclusion criteria during MM and BHs, acceptable data were collated in 10 participants (five women) classified as having OSA and in 14 control subjects (three women) for comparative analysis. Group mean values for translational and rotational movement during simulated apneas are presented in Table 2. High-quality mean voltage neurograms of multiunit MSNA were acquired in all 24 individuals. Characteristics of this subgroup of participants (Table 3) were broadly similar to and thus representative of the group as a whole (Table 1).

**TABLE 2:** Translational and rotational head movement during simulated apnea.

	Control (No or mild apnea)	OSA (Moderate–severe apnea)
Direction	*n* = 10	*n* = 14
X (mm)	0.97 ± 0.12	1.25 ± 0.14
Y (mm)	1.06 ± 0.16	1.27 ± 0.17
Z (mm)	2.29 ± 0.25	2.17 ± 0.20
Pitch (°)	2.36 ± 0.38	2.56 ± 0.29
Roll (°)	1.10 ± 0.16	1.11 ± 0.19
Yaw (°)	0.85 ± 0.16	0.96 ± 0.08

*Group mean values for translational (x, y, or z) and rotational (pitch, roll, or yaw) head motion (mean ± s.e) documented during Mueller maneuvers and breath-holds.OSA: obstructive sleep apnea.*

**TABLE 3:** Participant characteristics: subgroup meeting fMRI analysis criteria.

	Control (No or mild apnea)	OSA (Moderate or severe apnea)	
Characteristics	*n* = 10	*n* = 14	*p*-value
Age (years)	59 ± 9	57 ± 13	0.722
Men (*n*)	5	11	
AHI (events/h)	5 ± 3	34 ± 23	<0.001
BMI (kg/m^2^)	27 ± 3	31 ± 5	<0.05
MSNA (bursts/100 HB)	48 ± 17	66 ± 19	<0.05
MSNA (bursts/min)	27 ± 11	45 ± 14	<0.01
HR (bpm)	58 ± 11	65 ± 8	0.164
SBP (mmHg)	119 ± 9	127 ± 16	0.118
DBP (mmHg)	64 ± 10	71 ± 11	0.107
ODI	9 ± 9	31 ± 23	<0.01

*Mean ± SD.AHI, apnea–hypopnea index; BMI, body mass index; DBP, diastolic blood pressure; HB, heart beats; HR, heart rate; MSNA, muscle sympathetic nerve activity; ODI, oxygen desaturation index; OSA, obstructive sleep apnea; SBP, systolic blood pressure.*

### Physiological Responses to Simulated Apneas

**FIGURE 2:**
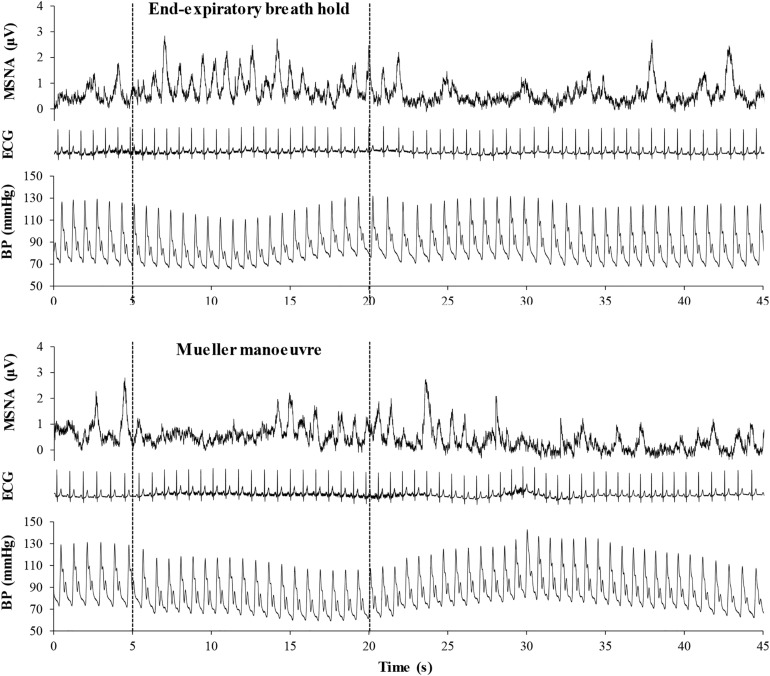
Mean voltage neurogram of muscle sympathetic nerve activity (MSNA), arterial blood pressure (BP), and the electrocardiogram (ECG), illustrating heart rate in a representative study participant before, during, and after a 15-s end expiratory breath-hold (*upper panel*) and a Mueller maneuver (*lower panel*). Note that the time delay between the initiation of the breathing task and the onset of sympathetic excitation was longer for the Mueller maneuver than for the breath-hold, due to initial aortic baroreceptor stimulation by the increase in intra-thoracic transmural pressure. However, the sympatho-excitatory responses to the two breathing tasks were of similar duration.

**FIGURE 3:**
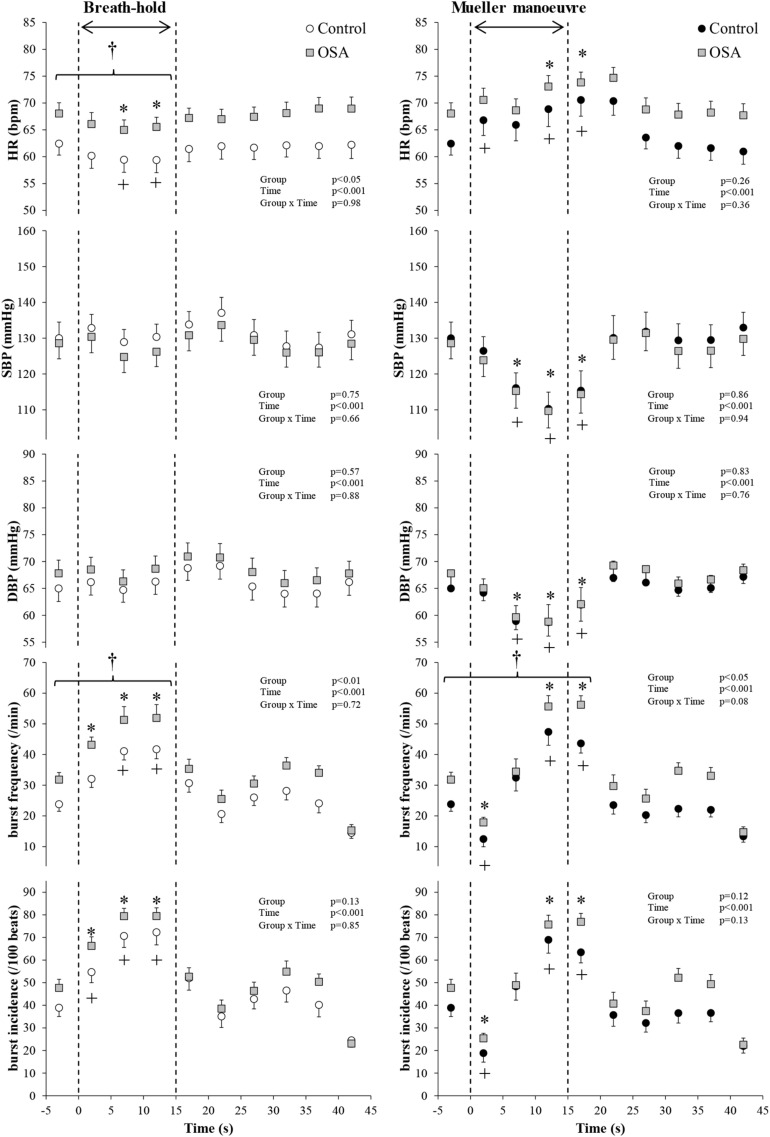
All study participants. Mean (±95% confidence limits) heart rate (HR), systolic and diastolic blood pressure (SBP and DBP), and muscle sympathetic nerve activity (MSNA) responses before, during, and after the series of 15 s of end-expiratory breath-holds (*left*) and 15 s of Mueller maneuvers (*right*) in 24 individuals with obstructive sleep apnea (OSA, *gray* squares) and 22 healthy controls (*white* and *black* circles) are displayed. The dashed vertical lines indicate the beginning (time = zero) and end (time = 15 s) of both maneuvers. Continuous responses were bin-averaged at 5-s intervals. For breath-holds, data during the 15 s of the task were compared with baseline values. For Mueller maneuvers, due to the time delay preceding sympathetic excitation, data during the 15 s of the task plus the first 5-s interval following the task were compared with baseline values. ^†^*p* < 0.05 compared to control group; ^∗^*p* < 0.05 compared to pre-maneuver (i.e., −2 s) in healthy controls; +*p* < 0.05 compared to pre-maneuver (i.e., −2 s) in OSA.

Figure 2 illustrates typical changes in MSNA, arterial blood pressure and HR of a participant before, during, and after a 15-s end expiratory BH, and a MM of similar duration. Figure 3, encompassing the entire study population, depicts mean changes in HR, blood pressure, and MSNA burst frequency and incidence for those with and without moderate to severe OSA. BHs and MMs had similar effects on HRs and blood pressures of these two cohorts. As anticipated (Bradley et al., 2003; Kimmerly et al., 2013), MSNA BI in both groups increased from baseline within the first 5 s of BHs (*p* < 0.001), reaching peak sympathetic excitation in the final 15 s (*p* < 0.001), and remained elevated until breathing resumed; thereafter, MSNA returned to baseline levels (*p* = 0.89). Consistent with previous observations demonstrating a reflex inhibitory response to acute stimulation of aortic baroreceptors by increased intra-thoracic transmural pressure (Bradley et al., 2003), BI fell (*p* < 0.005) within the first 5 s of the MM then returned to baseline levels at 10 s (*p* = 0.99) and rose above baseline during the final 15 s of MM (*p* < 0.005) and the first 5 s of recovery (*p* < 0.001). Although MSNA burst frequency was consistently higher, in participants with OSA, throughout both breathing tasks (*p* < 0.01 for BHs; *p* < 0.05 for MMs), the reflex sympathetic excitation induced by each task did not differ between those with OSA and the control participants (group × time interaction terms ≥0.08). Importantly, within this population as a whole, there were no between-group differences with respect to MSNA BI during either the BHs (*p* = 0.13) or the MMs (*p* = 0.12), and when expressed as BI, reflex sympathetic excitation induced by each task did not differ between those with OSA and the control participants (group × time interaction terms ≥0.13).

**FIGURE 4:**
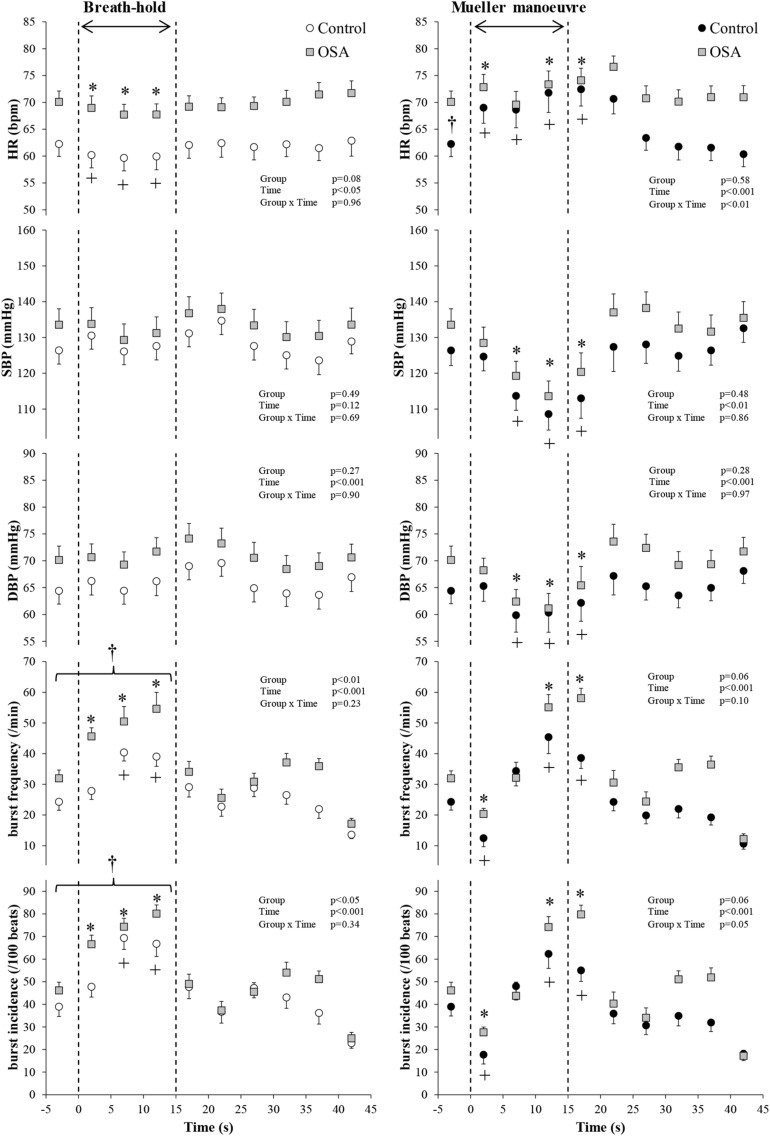
Participants with both blood oxygen level-dependent (BOLD) functional magnetic resonance signals meeting inclusion thresholds (see text) and high-quality mean voltage neurograms. Mean (±95% confidence limits) HR, SBP, DBP, and MSNA responses before, during, and after the series of 15 s of end-expiratory breath-holds (*left*) and 15 s of Mueller maneuvers (*right*) in 14 individuals with obstructive sleep apnea (OSA, *gray* squares) and 10 healthy controls (*white* and *black* circles) are displayed. The dashed vertical lines indicate the beginning (time = zero) and end (time = 15 s) of both maneuvers. Continuous responses were bin-averaged at 5-s intervals. For breath-holds, data during the 15 s of the task were compared with baseline values. For Mueller maneuvers, due to the time delay preceding sympathetic excitation, data during the 15 s of the task plus the first 5-s interval following the task were compared with baseline values. ^†^*p* < 0.05 compared to the control group; ^∗^*p* < 0.05 compared to pre-maneuver (i.e., −2 s) in healthy controls; ^+^*p* < 0.05 compared to pre-maneuver (i.e., −2 s) in OSA.

Figure 4 illustrates the changes in HR, blood pressure, and MSNA burst frequency and incidence of the study subjects with and without OSA whose movement artifact during simulated apnea did not exceed the defined exclusion thresholds. BHs and MMs had similar effects, in the two cohorts, on blood pressure (*p* ≥ 0.69), whereas it was higher in those with OSA HR over the course of the MM (*p* < 0.01 for group × time interaction). As with the participants as a whole, there were no between-group differences within this subset with respect to MSNA responses expressed as burst frequency during either the BHs (*p* = 0.23) or MMs (*p* = 0.10). When expressed as MSNA BI, responses to BHs in the two cohorts were similar (*p* = 0.34); there was a trend toward greater sympathetic excitation in those with OSA (*p* = 0.05).

Group mean BOLD fRMI image data were concordant with these neutral findings; second-level group comparison of MR images, synchronous with the peripheral neural responses depicted in Figure 4, detected nil after subtraction, indicating no between-group differences with respect to activity within cortical and cerebellar autonomic regions.

## Discussion

The acute, brief, mechanical, autonomic, chemical, and hemodynamic turbulence that is characteristic of obstructive apnea is exclusive to sleep, but when repeated nightly over months and years these induce an array of systemic after-effects evident during wakefulness (Floras, 2018; Taylor et al., 2018). One such consequence is long-term facilitation of central sympathetic outflow (Taylor et al., 2016a, 2018; Floras, 2018). The presumed stimulus to such autonomic neuroplasticity is recurrent hypoxia, which in experimental models increases the sensitivity of the afferent limb of the peripheral chemo-reflex to reductions in oxygen tension (Mahamed et al., 2005; Abboud and Kumar, 2014; Prabhakar, 2016); amplification of this neural input, if sustained, has been posited to induce trophic changes, such as the thickening documented, in patients with OSA, in cortical regions participating in cardiovascular autonomic regulation (Taylor et al., 2018), cognition, and other brain functions (Baril et al., 2017). Concurrently, hypoxia-mediated cellular trimming appears to thin other CAN regions, such as the left and right dorsal posterior insular cortices and the left posterior cingulate or precuneus (Taylor et al., 2018).

The present experiments considered an hypothesis inspired by three novel prior findings by our group. The first, involving healthy young volunteers invited to perform a volitional protocol replicated in the present series, was the identification of cortical and cerebellar regions participating in cardiovascular autonomic regulation in which BOLD contrast signaling either increased (activation) or decreased (deactivation) in temporal concordance with excitation of efferent post-ganglionic sympathetic discharge to skeletal muscle (Kimmerly et al., 2013). The second, revealed by a comparison of cortical autonomic regions of age-matched participants with and without polysomnography-classified OSA, was thinning of the left dorsal posterior insular cortex, proportionate to the ODI, and thickening of the left-mid cingulate cortex and thalami in those whose AHI was ≥15 events/h (Taylor et al., 2018). The third was the detection of positive correlations between thickening within these specific regions and muscle sympathetic BI, recorded during wakefulness (Taylor et al., 2018). The purpose of the present work was to test the hypothesis that individuals with documented OSA and such structural changes exhibit also greater muscle sympathetic nerve discharge and synchronous BOLD signal responses during volitional simulation of central or OSA by BHs and by MMs.

The results of these experiments do not support this hypothesis. Our principal findings with respect to MSNA and the concurrent BOLD contrast signal were both neutral and concordant; there were no between-group differences in response patterns of either variable to BHs or to MMs. These observations therefore are consistent with the results of prior dual-regression analyses, which found no between-group differences within this cohort with respect to the strength of resting-state connectivity within cortical and cerebellar CAN nodes of the salience network between those with moderate to severe OSA, relative to age- and sex-matched volunteers with no or only mild OSA (Taylor et al., 2016a).

A key strength of the present work is the classification of participants into cohorts with and without moderate to severe OSA on the basis of polysomnography, rather than unsubstantiated self-report. Indeed, several volunteers who professed normal breathing during sleep were discovered to have moderate or severe apnea; conversely, several who assumed that they did, because of loud snoring, had no or only mild airway obstruction during sleep. A second is the use of the MM, a stimulus to sympathetic activation that persists beyond its termination (Figure 2) (Bradley et al., 2003). This temporal discordance removes the potential confounding influences of volitional motor effort and baroreceptor- and chemoreflex-mediated, emotional, and sensory responses on cortical neural patterns generated by such simulation of obstructive apnea.

The MR incompatibility of the conventional microneurography apparatus deployed in our laboratory necessitated replication of these apnea-simulating tasks on different days; the assumption that the magnitude and duration of MSNA responses to apnea are consistent when repeated over such short time frame is reasonable (Kimmerly et al., 2004; Badrov et al., 2016). Using a pre-amplifier encased in stainless-steel Macefield’s group acquired 4-s neurograms interpolated between scanning periods; they identified, during spontaneous breathing, MSNA-coupled increases in BOLD signal intensity bilaterally, in the dorsolateral prefrontal cortex, posterior cingulate cortex, precuneus, ventromedial hypothalamus, and rostral ventrolateral medulla and on the left, within the mid-insular cortex and dorsomedial hypothalamus (Macefield and Henderson, 2019).

We considered several possible explanations for the absence of between-group differences. The duration of these volitional stimuli (15 s) was brief, relative to that of spontaneous apnea evident in the majority of patients with OSA (Bradley and Floras, 2003; Kasai et al., 2012), and thus may not have been sufficient to replicate the full intensity of sympathetic discharge elicited by the confluence of apnea, hypoxia, hypercapnia, and the arousal from sleep that terminates spontaneous obstructive or central events. In experiments by Morgan et al. (1993), supplemental oxygen (inspired fraction 0.30) attenuated markedly the time-dependent increases in MSNA, accompanied by 2% reductions in O_2_ saturation, elicited by BHs and MMs of greater duration (20 s). Also, patients with moderate to severe OSA will be exposed to scores or hundreds of apneic events over the course of the night, as compared with the six simulated events in the present protocol. Nonetheless, since longstanding OSA is accompanied by altered cortical autonomic gray matter thickness (Taylor et al., 2018) and, during wakefulness, both upward resetting of central sympathetic outflow (Taylor et al., 2016a, b) and augmented peripheral chemoreflex sensitivity (Mateika et al., 2004; Mahamed et al., 2005; Abboud and Kumar, 2014; Prabhakar, 2016), a single volitional apnea should be sufficient to detect between-group evidence for heightened central and peripheral autonomic responses to this stimulus–as has been shown, with MSNA recordings, for patients with heart failure (Bradley et al., 2003). For the purpose of between-cohort comparison, we classified participants conventionally, on the basis of their AHI, rather than the ODI (Linz et al., 2018). Importantly, in the present series, ODI tracked AHI and was three-fold higher in those with OSA. Present experiments were conducted during wakefulness, which resets upward, from sleep, the threshold eliciting PCO_2_-stimulated ventilation (Ainslie and Duffin, 2009) [and, by inference, chemo-reflex-mediated sympathetic discharge (Keir et al., 2019)]. Reflex sympathetic excitation would be anticipated earlier in the course of apneas occurring during sleep. An additional conjecture is that cortical autonomic neural activity in these study participants, who on average were twice as old as our previously studied healthy volunteers (Kimmerly et al., 2013), may be at or near ceiling under resting conditions. Thus, dynamic range constraint may preclude detection of material BOLD signal changes. In contrast to the stability of the head and neck of healthy young subjects when executing these instructed breathing maneuvers in our previous experiments (Kimmerly et al., 2013), and despite our training and rigorous attempts to ensure these middle-aged individuals could perform these interventions with minimal head movement, only 24 of our 46 participants (whose characteristics were similar to the group as a whole) were capable of doing so, suggesting subtle age-related sarcopenic loss of muscle strength. Such attrition raises the question as to whether subject numbers in this residual cohort were insufficient to detect between-group BOLD image differences. Notwithstanding, this principal study finding of neutrality was consistent across all endpoints measured: the hemodynamic and neural responses to both BHs and MMs were essentially identical in participants with and without OSA, within both the study population as a whole (Figure 3) and in the subgroup whose translational and rotational head motion permitted reliable image interpretation (Figure 4); consequently, if the MSNA responses were modulated by changes in neural activity within elements of the CAN, similar BOLD image data, as observed, would be anticipated. We nonetheless acknowledge that the range-of-motion artifact permitted for this subgroup analysis (about 2.2-mm motion and 2.5° rotation that is TASK correlated) may have been insufficiently conservative. Future investigations of this present pathophysiological construct in individuals with sleep-related breathing disorders should ensure a more robust head stabilization protocol.

In sum, the present investigation does not support the concept that recurring episodes of cyclical apnea during sleep alter cortical or peripheral neural responsiveness to their simulation during wakefulness by volitional MMs or BHs.

## Data Availability Statement

The raw data supporting the conclusions of this article will be made available by the authors, without undue reservation.

## Ethics Statement

The studies involving human participants were reviewed and approved by Research Ethics Board of the University Health Network. The patients/participants provided their written informed consent to participate in this study.

## Author Contributions

KT and JF contributed to the conception, design of the experiments, and secured grant funding. KT, NH, HM, and PM contributed to data collection. KT and DKe analyzed data and prepared figures. KT, DKe, and JF drafted and revised the manuscript. All authors contributed to the interpretation of data, approved the manuscript, and agreed to be accountable for all aspects of the work.

## Conflict of Interest

The authors declare that the research was conducted in the absence of any commercial or financial relationships that could be construed as a potential conflict of interest.
